# 797. Infection Prevention and Control Training Needs and Preferences Among Frontline Health Professionals

**DOI:** 10.1093/ofid/ofab466.993

**Published:** 2021-12-04

**Authors:** Sarah Stream, M Salman Ashraf, Nada Fadul, Dan K German, Mounica Soma, Kate Tyner, Nicolas W Cortes-Penfield, Nicolas W Cortes-Penfield

**Affiliations:** 1 Nebraska Medicine, Omaha, Nebraska; 2 University of Nebraska Medical Center, Omaha, NE

## Abstract

**Background:**

In 2020, the Nebraska Infection Control Assessment and Promotion program began collaborating with the Nebraska Department of Health and Human Services (NE DHHS) and the CDC to distribute infection prevention and control (IPC) training to frontline healthcare professionals (HCPs), focusing on nursing assistants (NAs), dentists, and other groups not traditionally targeted by IPC training. We conducted a learning needs assessment of these workers to plan high-yield curricula for each group.

**Methods:**

We distributed an online survey to Nebraska’s frontline HCPs via local professional society email lists and the NE DHHS’s weekly newsletter. The survey asked respondents to identify their professional role, practice setting (urban vs suburban vs rural), preferred sources and formats of training, and perceived need for additional training across multiple IPC topics.

**Results:**

456 HCPs completed our survey, including 177 NAs, 72 nurses, and 59 dentists; most HCPs practiced in a rural setting (62%). HCPs viewed the CDC as the most trustworthy source of IPC training (92% trusted, vs 71% for local health authorities, 64% for professional societies, and 43% for academic institutions); versus other respondents, NAs had substantially lower trust in all groups except the CDC. Respondents were more often interested in self-paced learning (63%) or interactive discussion with experts (53%) versus peer discussions (40%) or lectures (34%). Compared with other respondents, dentists were least interested in peer discussions (27%) and NAs in lectures (15%). Triage and screening was the only IPC training topic a majority of all respondents (51%) requested, though majorities of nurses (58%) and dentists (51%) also wanted training on environmental cleaning. Hand hygiene (12%) and personal protective equipment use (27%) were the least requested IPC topics, especially among NAs (5% and 18%).

**Conclusion:**

Nebraska’s frontline healthcare workers express high confidence in the CDC as a source of IPC training and prefer self-paced and expert discussion learning modalities. Key between-group differences indicate that individualizing curricula for NAs, dentists, and other HCPs may improve IPC training quality.

**Disclosures:**

**M. Salman Ashraf, MBBS**, **Merck & Co. Inc** (Grant/Research Support, I have recieved grant funding for an investigator initiated research project from Merck & Con. Inc. However, I do not see any direct conflict of interest related to the submitted abstract) **Nicolas W. Cortes-Penfield, MD**, Nothing to disclose

